# Structural Insights into the Methane-Generating Enzyme from a Methoxydotrophic Methanogen Reveal a Restrained Gallery of Post-Translational Modifications

**DOI:** 10.3390/microorganisms9040837

**Published:** 2021-04-14

**Authors:** Julia Maria Kurth, Marie-Caroline Müller, Cornelia Ulrike Welte, Tristan Wagner

**Affiliations:** 1 Department of Microbiology, Institute for Water and Wetland Research, Radboud University, Heyendaalseweg 135, 6525 AJ Nijmegen, The Netherlands; j.kurth@science.ru.nl (J.M.K.); c.welte@science.ru.nl (C.U.W.); 2 Microbial Metabolism Research Group, Max Planck Institute for Marine Microbiology, Celsiusstraße 1, 28359 Bremen, Germany; mmueller@mpi-bremen.de

**Keywords:** methyl-coenzyme M reductase, post-translational modifications, methoxydotrophic methanogenesis, X-ray crystallography, F_430_-cofactor, thermophilic archaeon

## Abstract

Methanogenic archaea operate an ancient, if not primordial, metabolic pathway that releases methane as an end-product. This last step is orchestrated by the methyl-coenzyme M reductase (MCR), which uses a nickel-containing F_430_-cofactor as the catalyst. MCR astounds the scientific world by its unique reaction chemistry, its numerous post-translational modifications, and its importance in biotechnology not only for production but also for capturing the greenhouse gas methane. In this report, we investigated MCR natively isolated from *Methermicoccus shengliensis*. This methanogen was isolated from a high-temperature oil reservoir and has recently been shown to convert lignin and coal derivatives into methane through a process called methoxydotrophic methanogenesis. A methoxydotrophic culture was obtained by growing *M. shengliensis* with 3,4,5-trimethoxybenzoate as the main carbon and energy source. Under these conditions, MCR represents more than 12% of the total protein content. The native MCR structure refined at a resolution of 1.6-Å precisely depicts the organization of a dimer of heterotrimers. Despite subtle surface remodeling and complete conservation of its active site with other homologues, MCR from the thermophile *M. shengliensis* contains the most limited number of post-translational modifications reported so far, questioning their physiological relevance in other relatives.

## 1. Introduction

**Figure 1:**
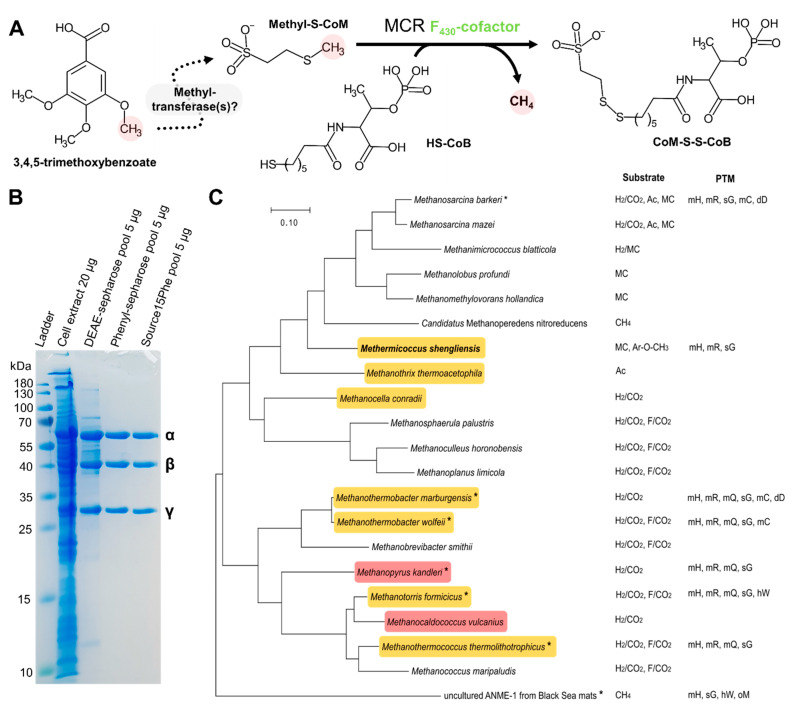
MCR metabolic function, purification, and phylogeny. (**A**) During methoxydotrophic growth, 3,4,5-trimethoxybenzoate (TMB) methyl-groups are transferred by an unknown mechanism to the central carbon metabolism of the methanogen. The methyl-group will be transferred onto HS-CoM and MCR will branch methyl-S-CoM to CoB-SH by a thiyl-radical based reaction catalyzed by its F_430_ cofactor. The end products of the reaction are methane and the heterodisulfide made of HS-CoM and CoB-SH. (**B**) Purification profile on SDS-PAGE of MCR α, β and γ subunits from TMB-grown cells. (**C**) Phylogenetic tree of concatenated MCR generated with MegaX using the Maximum Likelihood method and JTT matrix-based model (see Materials and Methods). Orange and red backgrounds indicate thermophiles and hyperthermophiles, respectively. Structural information exists for the species with asterisks. Post-translational modifications (PTM) observed in the structures are shown: mH, N^1^-methylhistidine; mR, 5(S)-methylarginine; mQ, 2(S)-methylglutamine; sG, thioglycine; mC, S-methylcysteine; dD, didehydroaspartate; hW, 6-hydroxytryptophan in *M. formicicus* and 7-hydroxytryptophan in ANME-1; oM, oxidized methionine. Growth substrates are also indicated (Ac, acetate; F, formate; MC, methylated compounds; Ar-O-CH_3_, methoxylated compounds).

Methanogenesis is a primitive energy metabolic pathway found only in the archaeal domain that evolved more than 3.46 Gyr ago [1,2]. During evolution, different types of methanogenesis arose, all of them sharing the common trait of releasing methane. Hydrogenotrophic methanogenesis reduces CO_2_ by using H_2_ or alternatively formate; aceticlastic methanogenesis disproportionates acetate in CO_2_ and CH_4_ and finally, methylotrophic methanogenesis uses methylated molecules such as methanol, methylamine(s), or methylsulfides [3,4]. In 2016, Mayumi and co-workers discovered a new methylotrophic pathway named methoxydotrophic methanogenesis in which the substrates are methoxylated aromatic compounds derived from lignin, oil, and coal [5]. The organism exhibiting this novel pathway is *Methermicoccus shengliensis*, a thermophilic archaeon that has been isolated from oil production water (75–80 °C) [6]. Methoxydotrophic methanogens such as *M. shengliensis* might play an important role in the carbon cycle of coal- and lignin-rich subsurface sediments as well as of oil reservoirs. These specialized methoxydotrophic methanogens are able to metabolize methoxy compounds intracellularly and transfer the methyl group on a carrier by a so far unknown mechanism (Figure 1A). As for all other methanogenic pathways, the methyl group must be transferred onto coenzyme M (HS-CoM) to be released as methane [4] by the Methyl-coenzyme M reductase (MCR).

MCR is a three-subunit complex harboring the cofactor F_430_, a nickel-containing corrinoid that gives its yellow color to the enzyme [7]. The chemical reaction catalyzed by the cofactor is a thiyl-radical mechanism in which the Ni(I)-active state will attack the thiol group of the methyl-S-CoM forcing the generation of methane and the formation of the heterodisulfide made of the HS-CoM and Coenzyme B (CoB-SH) [8]. Structural studies [9,10,11,12,13,14,15,16,17] revealed how MCR precisely coordinates the cofactor and coenzymes, and they also depicted a gallery of post-translational modifications that vary depending on the species [18]. Recent studies based on genetic manipulation of *Methanosarcina acetivorans* confirmed that thioglycine, S-methylcysteine and 5(S)-methylarginine are not required for catalysis [17,19,20] while the role of N^1^-methylhistidine is yet unknown [21]. Nevertheless, combinatorial interactions between modified residues were shown to alter the thermal stability of MCR as well as the growth fitness on different carbon sources [17]. It is assumed that these modifications might have a tuning-up function to improve the enzyme’s robustness under stress conditions and stimulate its turn-over [15,16]. Unfortunately, because of the high instability of the active Ni(I) state, enzymatic studies which characterize the impact of the loss of each modification are still a challenging task.

The overall reaction of methane generation by MCR is highly exergonic with a ∆*G*^0′^= −30 kJ/mol of methane formed [7]. Surprisingly, despite its thermodynamic difficulty, anaerobic methane oxidizers are using the reverse reaction to capture methane [14,22,23]. The methane activation by MCR-homologues highlights biotechnological potentials to mitigate the concentration of atmospheric methane [24], of which 50% worldwide is released by methanogens [1,25]. By domesticating the enzyme, it would be possible to trap and transform methane [26] or alternatively to block the methane release by inhibiting the enzyme [27]. Characterization of MCR from various methanogens is yielding an overview of the enzyme’s variability and provides templates for targeted mutagenesis.

The MCR from *M. shengliensis* (abbreviated as *Ms*MCR) is offering a new variation of the enzyme that might contain typical adaptations for methoxydotrophic growth in a high-temperature ecological niche. The structural features of *Ms*MCR presented in this report highlight a conserved active site with the lowest post-translational modification content reported so far.

## 2. Materials and Methods

### 2.1. Phylogenetic Analyses

Protein sequences used for phylogenetic analyses are, organized by organism (α,β,γ): *Methermicoccus shengliensis* DSM 18856 (WP_084174107.1, WP_042686194.1, WP_042686201.1), *Methanosarcina barkeri* Fusaro (WP_011305916.1, WP_011305920.1, WP_011305917.1), *Methanosarcina mazei* Go1 (WP_011033189.1, WP_011033193.1, WP_048045871.1), *Methanothrix thermoacetophila* PT (WP_011695757.1, WP_011695760.1, WP_011695758.1), *Candidatus* Methanoperedens nitroreducens ANME-2d (WP_048089615.1, WP_048089608.1, WP_048089613.1), *Methanolobus profundi* Mob M (WP_091936029.1, WP_091936035.1, WP_091936030.1), *Methanomethylovorans hollandica* DSM 15978 (WP_015325028.1, WP_015325024.1, WP_015325027.1), *Methanimicrococcus blatticola* DSM 13328 (WP_133517056.1, WP_133517053.1, WP_133517055.1), *Methanotorris formicicus* Mc-S-70 (WP_007043982.1, WP_007043986.1, WP_007043983.1), *Methanothermococcus thermolithotrophicus* DSM 2095 (WP_018153522.1, WP_018153526.1, WP_018153523.1), *Methanothermobacter marburgensis* strain Marburg type I (WP_013296337.1, WP_013296341.1, WP_013296338.1), *Methanopyrus kandleri* AV19 (WP_011019025.1, WP_011019021.1, WP_011019024.1), *Methanothermobacter wolfeii* isolate SIV6 (SCM58307.1, SCM58314.1, SCM58308.1), *Methanosphaerula palustris* E1-9c (WP_012618913.1, WP_012618909.1, WP_012618912.1), *Methanoculleus horonobensis* JCM 15517 (WP_067078350.1, WP_067078343.1, WP_067078348.1), *Methanoplanus limicola* DSM 2279 (WP_004079635.1, WP_004079639.1, WP_004079636.1), *Methanocella conradii* isolate 1 (WP_174590719.1, WP_174590722.1, WP_014405505.1), *Methanococcus maripaludis* C7 (WP_011977191.1, WP_011977187.1, WP_011977190.1), *Methanocaldococcus vulcanius* M7 (WP_012819563.1, WP_012819559.1, WP_012819562.1), *Methanobrevibacter smithii* DSM 2375 (WP_019262578.1, WP_004035807.1, WP_004035804.1), ANME-1 from Black Sea mats, Uncultured archaeon ANME-1 (D1JBK4, D1JBK2, D1JBK3). The sequences were aligned by using the Clustal W tool in MegaX [28] followed by the evolutionary analyses conducted with the same software. The evolutionary history was inferred using the Maximum Likelihood method and JTT matrix-based model [29]. The tree with the highest log likelihood (−27,171.46) is shown. Initial tree(s) for the heuristic search were obtained automatically by applying Neighbor-Join and BioNJ algorithms to a matrix of pairwise distances estimated using the JTT model, and then selecting the topology with superior log likelihood value. The tree is drawn to scale, with branch lengths measured in the number of substitutions per site. This analysis involved 21 amino acid sequences. There were a total of 1328 positions in the final dataset.

The references regarding the substrate utilization for each methanogen and MCR post-translational modifications, presented in the phylogenetic tree, can be found in the supplemental information.

### 2.2. Cultivation of Methermicoccus Shengliensis

*Methermicoccus shengliensis* ZC-1 (DSM 18856) [6] was obtained from the DSMZ (Braunschweig, Germany) and cultivated in modified DSM medium 1084. Sludge fluid was replaced by trace element solution (100 × trace element solution: 1.5 g/L nitrilotriacetic acid, 3 g/L MgSO_4_∙7 H_2_O, 0.45 g/L MnSO_4_∙2 H_2_O, 1 g/L NaCl, 0.1 g/L FeSO_4_∙7 H_2_O, 0.18 g/L CoSO_4_∙6 H_2_O, 0.1 g/L CaCl_2_∙2 H_2_O, 0.18 g/L ZnSO_4_∙7 H_2_O, 0.01 g/L CuSO_4_∙5 H_2_O, 0.02 g/L KAl(SO_4_)_2_∙12 H_2_O, 0.01 g/L H_3_BO_3_, 0.01 g/L Na_2_WO_4_∙2 H_2_O, 0.01 g/l Na_2_MoO_4_∙2 H_2_O, 0.025 g/L NiCl_2_∙6 H_2_O, 0.01 g/L Na_2_SeO_3_) and vitamin solution (1000 × vitamin solution: 20 mg/L biotin, 20 mg/L folic acid, 100 mg/L pyridoxine-HCl, 50 mg/L thiamin-HCl∙2 H_2_O, 50 mg/L riboflavin, 50 mg/L nicotinic acid, 50 mg/L D-Ca-pantothenate, 2 mg/L vitamin B_12_, 50 mg/L p-aminobenzoic acid, 50 mg/L lipoic acid). The amount of supplied coenzyme M was reduced 20-fold (0.13 g/L) and 2.5 g/L NaHCO_3_ instead of 1 g/L Na_2_CO_3_ was used. The medium was sparged with N_2_:CO_2_ in an 80:20 ratio before autoclaving. 10 mM 3,4,5-trimethoxybenzoate was used (0.5 M stock solution was prepared by adjusting the pH to 8). Methanol-grown cultures were provided with 100 mM methanol instead of TMB. The cultures were incubated at 65 °C. *M. shengliensis* cells were harvested anaerobically (10,000× *g*, 25 min and 4 °C) after reaching the late exponential phase and cells were frozen anaerobically at −80 °C.

### 2.3. Native Purification of MsMCR

About 6 g of cells were defrosted while gassing for 10 min with N_2_ gas. Afterwards, cells were resuspended in 15 mL anaerobic IEC buffer (50 mM Tris/HCl pH 8, 2 mM dithiothreitol (DTT)), sonicated (6 × 75% amplitude for 10 s with 20 s break, Bandelin sonopuls, Berlin, Germany), centrifuged (16,250× *g*, 30 min at room temperature) and the supernatant was collected. The pellets were resuspended in 15 mL anaerobic IEC buffer, sonicated (5 × 75% amplitude for 10 s with 20 s break), centrifuged (16,250× *g*, 30 min) and the supernatant was combined with the supernatant from the previous step. The supernatant was then diluted 5-fold with IEC buffer, filtered through a 0.2 μm filter (Sartorius, Göttingen, Germany) and loaded on a 15 mL DEAE column (GE healthcare, Chicago, IL, USA). Proteins were eluted by applying a 0 to 0.45 M NaCl gradient, over 120 min with a flow rate of 2 mL/min. Under these conditions, MCR eluted between 0.28 and 0.33 M NaCl. The fractions containing MCR were pooled, diluted with 4 volumes of HIC buffer (25 mM Tris/HCl pH 7.6, 2 mM DTT, 2 M (NH_4_)_2_SO_4_), filtered through a 0.2 μm filter and loaded on a 5 mL phenyl sepharose column (GE healthcare). Proteins were eluted by applying a 1.7 to 0 M gradient of (NH_4_)_2_SO_4_ over 60 min with a flow rate of 1 mL/min. MCR was eluting between 1.25 and 1 M of (NH_4_)_2_SO_4_. Pooled MCR fractions were diluted with 4 volumes of HIC buffer, filtered through a 0.2 μm filter and loaded on a Source15Phe 4.6/100 PE column (GE healthcare). Proteins were eluted by applying a gradient of 1.6 to 0 M (NH_4_)_2_SO_4_, over 60 min with a flow rate of 1 mL/min. Fractions of apparently pure MCR were eluting between 1.45 and 1.2 M (NH_4_)_2_SO_4_. Pooled fractions were concentrated with 15 mL Millipore Ultra-10 centrifugal filter units (Merck, Darmstadt, Germany) and the buffer was exchanged for storage buffer (25 mM Tris pH 7.6, 10% *v/v* glycerol, 2 mM DTT). MCR was concentrated to 47 g/L and immediately used for crystallization and spectrophotometry. Protein concentration was evaluated by the Bradford method according to manufacturer (Bio-Rad, Hercules, CA, USA) recommendations. MCR from methanol-grown cells was purified following a similar protocol consisting of DEAE and Phenyl sepharose.

To compare the Stokes radius of *Ms*MCR purified from methanol and TMB-grown cells, both proteins (0.55 mg of purified *Ms*MCR) were injected on a Superdex 200 10/300 Increase GL (GE Healthcare) at a flow rate of 0.4 mL/min at 20 °C. Both *Ms*MCRs showed an elution volume of 10.55 mL.

### 2.4. High-Resolution Clear Native (hrCN) Polyacrylamide Gel Electrophoresis (PAGE)

The hrCN-PAGE protocol was adapted from Lemaire et al. [30]. Glycerol (20% *v/v* final) was added to samples and 0.001% *w/v* Ponceau S was used as a protein migration marker. The electrophoresis cathode buffer contained a buffer mixture of 50 mM Tricine; 150 mM Bis-Tris pH 7 supplemented with 0.05% *w/v* sodium deoxycholate; 0.01% *w/v* dodecyl maltoside. The anode buffer contained 150 mM Bis-Tris buffer, pH 7. The NativeMark™ unstained protein standard from Thermo Fisher Scientific (Darmstadt, Germany) was used as a ladder. hrCN-PAGE were carried out using an 8 to 15% linear polyacrylamide gradient, gels were run with a constant 20 mA current using a PowerPac^TM^ Basic Power Supply (Bio-Rad). After electrophoresis, the protein bands were stained with Instant Blue^TM^ (Expedeon, Heidelberg, Germany).

### 2.5. Mass Spectrometry

MCR α-subunit was identified with help of matrix assisted laser desorption/ionization time-of-flight mass spectrometry (MALDI-TOF MS) by the following protocol. Protein bands were cut into small pieces (about 3 × 3 mm) and destained by adding the following solvents/buffers successively: 20 µL acetonitrile (ACN), 20 µL 50 mM ammonium bicarbonate (ABC) buffer, 50% *v/v* ACN in ABC buffer and 20 µL ACN. After each addition, samples were swirled and incubated for 10 min at room temperature (RT) followed by removing the liquid from the sample. Those steps were repeated until the gel pieces were destained. For reduction and alkylation, samples were incubated in 20 µL 10 mM DTT at 56 °C for 30 min, the liquid removed, and the following solvents/buffers successively added: 20 µL ACN, 20 µL 50 mM 2-chloroacetamide in 50 mM ABC buffer, 20 µL ACN, 20 µL ABC buffer, 20 µL ACN, and 20 µL ABC buffer. After each addition, samples were incubated for 10 min at RT followed by removing the liquid from the sample. For trypsin digestion, 10 µL of 5 ng/µL trypsin (V5518, Promega, Madison, WI, USA) in 50 mM ABC buffer were added to the gel pieces followed by 30 min incubation at RT. Afterwards 20 µL ABC buffer were added and the samples were incubated overnight at 37 °C. The samples were sonicated for 20 s in a sonication bath (Branson 2510, Brookfield, CT, USA) and 20 µL 0.1% *v/v* trifluoroacetic acid were added. The samples were incubated for 20 min at RT before the extract liquid was transferred to a new tube. 20 µL ACN were added to the remaining trypsin digests, the samples were incubated for 30 min at RT before the extract liquid was combined with the extract liquid from before. The samples were then dried in a Sanvant ISS110 speedVac (Thermo Scientific, Waltham, MA, USA) until ~5 µL remained. Then, 0.5 µL of the extracted peptides was pipetted on a MALDI-TOF sample plate and directly mixed with an equal volume of matrix solution containing 10 mg/mL α-cyano-4-hydroxy-α-cyanocinnamic acid in 50% *v/v* ACN/0.05% *v/v* trifluoroacetic acid. After drying of the sample this process was repeated once more. A spectrum in the range of 600 to 4000 *m/z* was recorded using a Microflex LRF MALDI-TOF (Bruker). The Biotools software (Bruker Life Sciences) was used to perform a MASCOT search (Matrix Science Ltd., London, UK) by using the *M. shengliensis* protein database (GenBank accession number NZ_JONQ00000000.1). Search parameters allowed a mass deviation of 0.3 Da, one miscleavage, a variable modification of oxidized methionines and a fixed modification of carbamidomethylated cysteines. The N^1^-methylhistidine275 containing peptide (1452.9 Da vs predicted mass of 1452.7 Da) and the Gln418 containing peptide (3468.8 Da vs predicted mass of 3468.6 Da) were detected (see Supplemental Figure S1A). The mass of the 5(*S*)-methylarginine289, containing peptide is below the 600 m/z threshold and was therefore not detected. The alkylated peptide containing the thioglycine463, aspartate468 and cysteine470 (LGFF**G**YDLQ**D**Q**C**GAANVFSYQSDEGLPLELR) was observable (3524.7 Da vs predicted mass of 3525.6 Da; Supplemental Figure S1A,B) at a signal over noise threshold of 1.9 and the mass fits in the 1 Dalton range.

### 2.6. Crystallization

MCR crystals were obtained aerobically by initial screening at 18 °C using the sitting drop method on 96-Well MRC 2-Drop Crystallization Plates in polystyrene (SWISSCI). The crystallization reservoir contained 90 µL of the following crystallization condition: 25% *w/v* polyethylene glycol 3350, 100 mM Bis-Tris pH 5.5, and 200 mM lithium sulfate. The crystallization drop contained a mixture of 0.6 µL *Ms*MCR at a concentration of 47 mg/mL and 0.6 µL of the crystallization condition. Thick yellow brick-shaped crystals appeared within two weeks.

### 2.7. X-ray Data Collection and Model Refinement/Validation

All X-ray crystallographic data and refinement statistics are presented in Table 1. MCR crystals were soaked in the crystallization solution supplemented with 20% *v/v* glycerol for 6 s before being transferred to liquid nitrogen. All diffraction experiments were performed at 100 K on Proxima-1 beamline, SOLEIL synchrotron, Saclay, France. The data were processed with xdsme and scaled with SCALA from the CCP4 package [31]. *Ms*MCR structure was solved by molecular replacement with Phenix [32] using MCR from *Methanosarcina barkeri* (PDB 1E6Y [13]) as a template. The model was manually built via Coot [33] and refined with BUSTER [34] by using the non-crystallographic symmetry and translational-liberation screw (TLS). The last refinement steps were performed with hydrogens in riding position. The model was ultimately validated by the MolProbity server [35] (http://molprobity.biochem.duke.edu, accessed on 15 of February 2021). Hydrogens were omitted in the final deposited model (PDB code 7NKG). All figures were generated and rendered with PyMOL (V. 1.8, Schrödinger, LLC).

## 3. Results

### 3.1. Purification and Crystallization of MsMCR Obtained under Methoxydotrophic Methanogenesis

The cell extracts of *M. shengliensis* grown with 3,4,5-trimethoxybenzoate or methanol as the main carbon and energy source, were first compared and showed a similar profile for the three subunits constituting MCR (Supplemental Figure S2A). McrA identification was confirmed by MALDI-TOF MS with a molecular weight search (MOWSE) score of 97 and an amino acid sequence coverage of 43%. MCR was anaerobically purified to homogeneity by anionic exchange and hydrophobic interaction chromatography (Figure 1B and Supplemental Figure S2B,C), yielding 43.6 mg of purified protein (see Materials and Methods) that corresponds to 12% of the total protein extract. It is generally assumed that MCR is catalyzing the rate-limiting step of methanogenesis and methanogens maintain their high-flux metabolism by expressing enormous amounts of the enzyme [7].

The purified MCR, containing an equal stoichiometry of the three subunits, has a characteristic yellow color coming from its F_430_-cofactor. The UV/Visible spectra (Supplemental Figure S2D) is typical of the Ni(II) red1-silent state with an absorption peak at 424 nm [36]. O_2_-incubation for one hour did not modify the spectra and therefore the sample was crystallized aerobically.

### 3.2. A Conserved Overall Structure and Active Site

**Figure 2:**
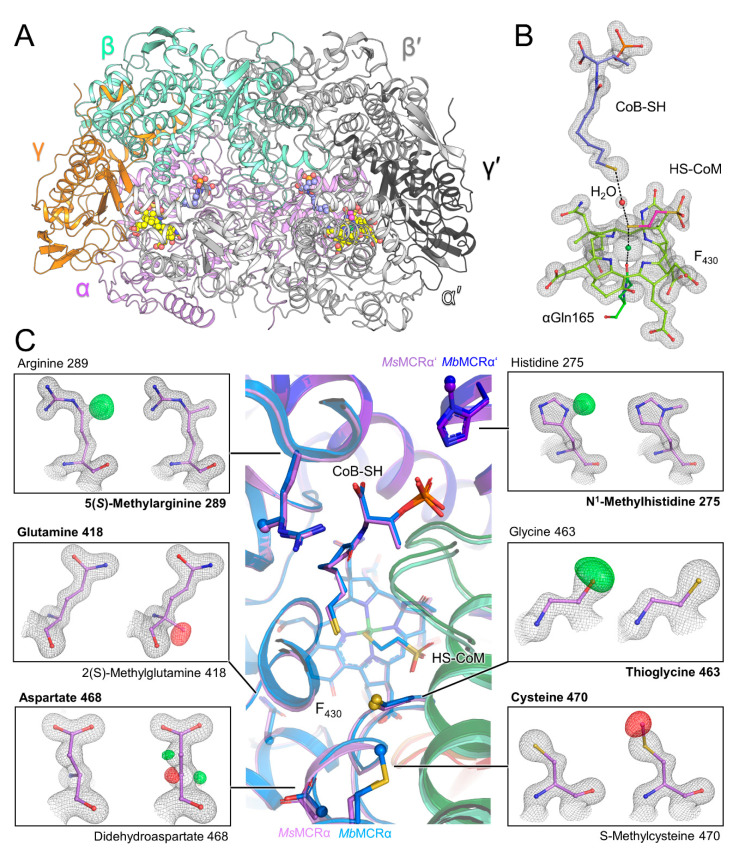
*Ms*MCR structure and its post-translational modifications. (**A**) *Ms*MCR (αβγ)_2_ organization with each chain colored differently. F_430_, HS-CoM and CoB-SH are in balls and sticks and colored in yellow, pink, and light blue, respectively. (**B**) Close up of the active site. 2*F*_o_-*F*_c_ electron density map for the F_430_ and coenzymes is contoured at 2-σ. (**C**) Superposition of *Ms*MCR (same color code as panel A) on *Mb*MCR (α, β, γ in blue, dark green and red respectively). The ligands and modified residues are in balls and sticks with the modifications as spheres. Each panel presents the 2*F*_o_-*F*_c_ map contoured at 2-σ (black mesh) and the *F*_o_-*F*_c_ map contoured at 4-σ (green, positive, and red, negative) after refinement for a classic (left) or modified (right) residue. Final modelled residue is highlighted in bold.

X-ray diffraction measurements were performed on *Ms*MCR crystals and revealed a primitive orthorhombic crystalline form. The structure of MCR from *Methanosarcina barkeri* (*Mb*MCR) was used for molecular replacement based on a phylogenetic analysis (Figure 1C) and the *Ms*MCR structure was refined to 1.6-Å resolution. The asymmetric unit contains two dimers with the typical (αβγ)_2_ organization (Figure 2A). Interestingly, while the first dimer shows an excellent fit in the electron density (average B-factor = 22.6 Å^2^), the second dimer has a very high average B-factor (51.3 Å^2^), which made its accurate modelling challenging. All following analyses were therefore performed on the first stable dimer. The model was compared with three homologues: the terrestrial mesophile *M. barkeri* (optimal growth temperature of 35 °C, PDB code 1E6Y), the terrestrial thermophile *Methanothermobacter marburgensis* (optimal growth temperature of 65 °C, *Mm*MCR type I PDB code 5A0Y), and the marine thermophile *Methanothermococcus thermolithotrophicus* (optimal growth temperature of 65 °C, *Mt*MCR PDB code 5N1Q). It is worth noting that *M. marburgensis* and *M. thermolithotrophicus* are hydrogenotrophic methanogens growing at the same temperature as *M. shengliensis*. *Ms*MCR and its structural homologues aligned very well with a root mean square deviations below 1-Å for the three different chains (Supplemental Table S1). This is not surprising considering the high sequence identity between the four MCRs (Supplemental Table S1). The extended loop following the N-terminal helix of the α-subunit found in *Mb*MCR (residues 18–29) is also conserved in *Ms*MCR (residues 19–33) and might have a stabilizing role. Only one discrepancy was noticeable, the loop 53–66 of the β-subunit is shifted in one of the monomers (Supplemental Figure S3A,B). Since the loop is on the surface, distant from the active site and involved in a crystallographic contact, this shift was most probably due to a packing artefact rather than a typical trait for this family. An inspection of the electrostatic charge profile on the proteins surface reflects the classic positively charged entrance of the CoB-SH channel. The electrostatic charge repartition of *Ms*MCR fits very well with the one from *Mb*MCR rather than the two other thermophiles (Supplemental Figure S3C–F) showing that thermophilic and high salt adaptations of *M. shengliensis* have not drastically modified the enzyme surface.

The active site is identical compared to the three other structural homologues with the same coordination of the coenzymes and F_430_-cofactor (Figure 2B). Both coenzymes are bound at very high occupancy with a distance of 6.2 Å separating their sulphur groups as previously seen in other Ni(II) red1-silent structures [10,11,13,15,16], the density between the thiol groups was interpreted as a water molecule. The well-defined electron density for the F_430_ perfectly fits the classic cofactor observed in structural homologues (Figure 2B). The HS-CoM has an average B-value of 21.6 Å^2^ that is 4.1 Å^2^ higher than CoB-SH in the most defined *Ms*MCR dimer This could come from a higher vibration or slightly lower occupancy of the HS-CoM already seen in MCR structures [15].

### 3.3. The Smallest Post-Translational Modification Gallery Observed in Methanogens

The high quality of the obtained electron density map confirmed the presence of three modified residues: N^1^-methylhistidine275, 5(*S*)-methylarginine289, and the thioglycine463. The calculation of an omit map for the three modifications unambiguously confirmed this result (Figure 2C). Surprisingly, the S-methylcysteine and didehydroaspartate found in the close relatives *M. barkeri* [13,18] and *Methanosarcina acetivorans* [17] are not detected in *M. shengliensis*. After forcing the modelling of a didehydroaspartate at position 468 and S-methylcysteine 470 in *M. shengliensis* the resulting 2*F*_o_-*F*_c_ and *F*_o_-*F*_c_ maps post-refinement confirmed the absence of both modifications (Figure 2C). With help of MALDI-TOF MS analysis we were able to detect the additional methylation in the peptide containing the His275 (1452.9 Da vs predicted mass of 1452.7 Da). We also observed the peptide containing the thioglycine463, however with a low signal/noise ratio of 1.9 (see Materials and Methods). The tryptic digestion and MALDI-TOF MS analysis could not detect the peptide containing the methylated Arg289 due to its small size.

Asp468 and Cys470 in *Ms*MCR present a similar position and coordination compared to the modified versions in *Mb*MCR and *Mm*MCR and no mutation in the direct surrounding appeared to counterbalance the absence of modifications (Supplemental Figure S4A). Rather subtle readjustments take place, such as shorter hydrogen bond distances, which might ultimately affect the loops coordinating the coenzymes as previously hypothesized for *Mm*MCR and MCR from *Methanothermobacter wolfeii* [15,16].

Methanobacteriales, Methanococcales, *Methanopyrus kandleri,* and *Methanoculleus thermophilus* [18] contain a 2(S)-methylglutamine close to the F_430_. This modification is not present in *Mb*MCR and in *M. acetivorans* and the presented structural data shows a classic glutamine at this position in *Ms*MCR (Figure 2C). A water molecule “fills” the absence of the methylation that might indirectly stabilize the F_430_ position via the αTyr350 (Supplemental Figure S4B). The peptide containing a classic Gln418 in *Ms*MCR was also detected by mass spectrometry (see Materials and Methods).

Surprisingly, these results reveal that despite the high similarity in sequence and structure, *Ms*MCR operates with a reduced gallery of post-translational modifications compared to *M. barkeri* or *M. acetivorans*.

## 4. Discussion

Anaerobic archaea have already been thriving on earth for billions of years and developed a variety of metabolic pathways to utilize a broad range of substrates. Methanogens living in deeper sediment layers managed to utilize coal, oil, and lignin derivatives as methyl donors, which provide methanogens with an abundant source of carbon and energy. This process could be an inspiration to transform methoxy-compounds to methane and use it as biofuel. *M. shengliensis* represents an excellent model organism to study this new pathway in depth, as its enzymes, involved in the methoxydotrophic metabolism are accessible for biochemical characterization. In this work, we isolated and structurally characterized the enzyme involved in the methane release, one of the last reactions of methoxydrotrophy. Under methoxydotrophic growth conditions, *M. shengliensis* contains a tremendous amount of MCR as also found in methanol-grown cultures (Supplemental Figure S2A).

*M. shengliensis* contains the different required machineries to feed on a broad variety of substrates such as methanol, methylamine(s), and different methoxylated aromatic compounds [5,6]. It was suggested that the post-translational modifications of the MCR from *M. acetivorans* might affect its growth robustness when grown on different substrates [17]. The systematic or complete deletion of the three genes involved in the arginine and cysteine methylation, as well as thioglycine formation, have indeed some impact on the growth when the methanogen uses different carbon sources [17]. Based on these results, it can be assumed that the acquisition of the methoxydotrophic pathway will favor the apparition of additional modifications or adaptive traits. However, instead of harboring new modified residues, *Ms*MCR shows a reduced set of modifications, which was unexpected. Only the core modifications methyl-histidine, methyl-arginine, and thioglycine are present.

The absence of S-methylcysteine in *Ms*MCR is explained by the fact that the gene coding for the methyl-transferase responsible of its installation (MA_RS23695 in *M. acetivorans*) is absent in the current genome of *M. shengliensis* strain DSM 18856 (Assembly number GCA_000711905.1). No conclusions can be drawn for the didehydroaspartate and methylglutamine since the biosynthetic machineries are still unknown. However, the genes coding for the enzyme responsible of the thioamidation (YcaO coding gene: BP07_RS07665 and TfuA coding gene: BP07_RS07670) of αGly463 and methylation of the αArg289 (coding gene: *mmp10*) are present in the *M. shengliensis* genome, which allow the installation of these modifications as observed in the *Ms*MCR structure.

Despite their different ecological niches, *Ms*MCR and *Mb*MCR share a remarkably similar organization and electrostatic surface, illustrating the close relationship between the two organisms in accordance with the phylogenetic studies (Figure 1C). The active site is identical to already described MCRs. Such perfect conservation, even the one from anaerobic methane oxidizers, such as marine ANME-1 clade archaea [14], depicts how challenging the chemical mechanism of methane generation/capture is. As always, in the absence of an active structure of MCR Ni(I) state, it is difficult to derive final conclusions on the possible structural roles of modifications during the catalysis.

Although the active site of different MCRs is quite conserved, the overall amino acid sequence of MCR enzymes from various archaea differs (Figure 1C) and notable differences exist between the structurally characterized MCRs and *Ms*MCR. Interestingly, *Ms*MCR is closely related to MCR from *Candidatus* Methanoperedens nitroreducens, which is an anaerobic methane oxidizer of the ANME-2d clade. This indicates that the MCR enzymes of some methane producing, and methane consuming, archaea might not only be very similar regarding their active site, but most likely also regarding overall structural features. The structural information we gained on *Ms*MCR might therefore be useful to understand MCR enzymes from ANME-2 archaea better.

To conclude, accumulating structural information from metabolically and ecologically diverse MCRs is broadening our scope on their natural diversity, as well as their post-translational modification repertoire. The latter is not following phylogenetic relationships or consistency regarding the growth conditions (e.g., temperature, and salt concentration) and it is still a mystery why such energy-extremophiles sacrifice cellular energy to install MCR post-translational modifications. The synergistic effort of genetic modification by using *M. acetivorans* as chassis and the exploration of the broad natural MCR landscape will hopefully provide more clues to further investigate the function of the modifications and could ultimately improve the robustness of the biotechnological application of MCRs.

## Figures and Tables

**Table 1:** X-ray crystallographic data and refinement statistics.

	MCR from *M. shengliensis*
**Data collection**	
Wavelength (Å)	0.97856
Space group	*P*2_1_2_1_2_1_
Resolution (Å)	49.41–1.60 (1.69–1.60)
Cell dimensions: a, b, c (Å)	132.62 148.18 235.41
R_merge_ (%) ^a^	9.1 (121.6)
R_pim_ (%) ^a^	5.1 (66.1)
CC_1/2_^a^	0.997 (0.356)
I/σ*_I_* ^a^	8.3 (1.0)
Completeness ^a^	99.7 (99.3)
Redundancy ^a^	4.2 (4.3)
Number of unique reflections ^a^	602614 (87124)
**Refinement**	
Resolution (Å)	48.36–1.60
Number of reflections	602,442
R_work_/R_free_^b^ (%)	0.1725/0.1904
Number of atoms	
Protein	38,087
Ligands/ions	405
Solvent	4298
Mean B-value (Å^2^)	35.0
Molprobity clash score, all atoms	0.67
Ramachandran plot	
Favored regions (%)	97.71
Outlier regions (%)	0.16
Rmsd ^c^ bond lengths (Å)	0.007
Rmsd ^c^ bond angles (°)	0.95
**PDB ID code**	7NKG

^a^ Values relative to the highest resolution shell are within parentheses. ^b^ R_free_ was calculated as the R_work_ for 5% of the reflections that were not included in the refinement. Refined model contained hydrogens. ^c^ rmsd, root mean square deviation.

## Data Availability

The structure was deposited in the protein data bank under the ID: 7NKG.

## Supplementary Materials

The following are available online at https://www.mdpi.com/article/10.3390/microorganisms9040837/s1, Figure S1: Mass spectrometry data obtained on MCRα peptides, Figure S2: Native PAGE and UV/visible spectra profile of purified *Ms*MCR, Figure S3: Structural and electrostatic charge differences between *Ms*MCR and its homologues, Figure S4: Close up of the environment at the expected modified residues didehydroaspartate, S-methylcysteine and 2(S)-methylglutamine in *Ms*MCR, Table S1: Sequence identity between the subunits of different MCRs and root mean square deviation (r.m.s.d.) of structurally characterized MCRs.
