# Supplementary material for: Structural Insights into the Methane-Generating Enzyme from a Methoxydotrophic Methanogen Reveal a Restrained Gallery of Post-Translational Modifications
Source: Microorganisms. 2021 Apr 14;9(4):837. doi: 10.3390/microorganisms9040837 (PMC8070964; doi:10.3390/microorganisms9040837)
Supplement: Supplementary file 1 [file microorganisms-09-00837-s001.pdf]

**Structural insights into the methane-generating enzyme from a methoxydotrophic methanogen reveal a restrained gallery of post-translational modifications**

Julia Maria Kurth[1#], Marie-Caroline Müller[2#], Cornelia Ulrike Welte[1] and Tristan Wagner[2*]

**Supplementary files.**

Supplemental Figure 1. Mass spectrometry data obtained on MCRα peptides.

Supplemental Figure 2. Native PAGE and UV/visible spectra profile of purified *Ms*MCRs.

Supplemental Figure 3. Structural and electrostatic charge differences between *Ms*MCR and its homologues.

Supplemental Figure 4. Close up of the environment at the expected modified residues didehydroaspartate, S-methylcysteine and 2(S)-methylglutamine in *Ms*MCR.

Supplementary Table 1. Sequence identity between the subunits of different MCRs and root mean square deviation (r.m.s.d.) of structurally characterized MCRs.

References regarding the substrate utilisation of methanogens and MCR post-translational modifications used in the phylogenetic tree (Fig. 1C).

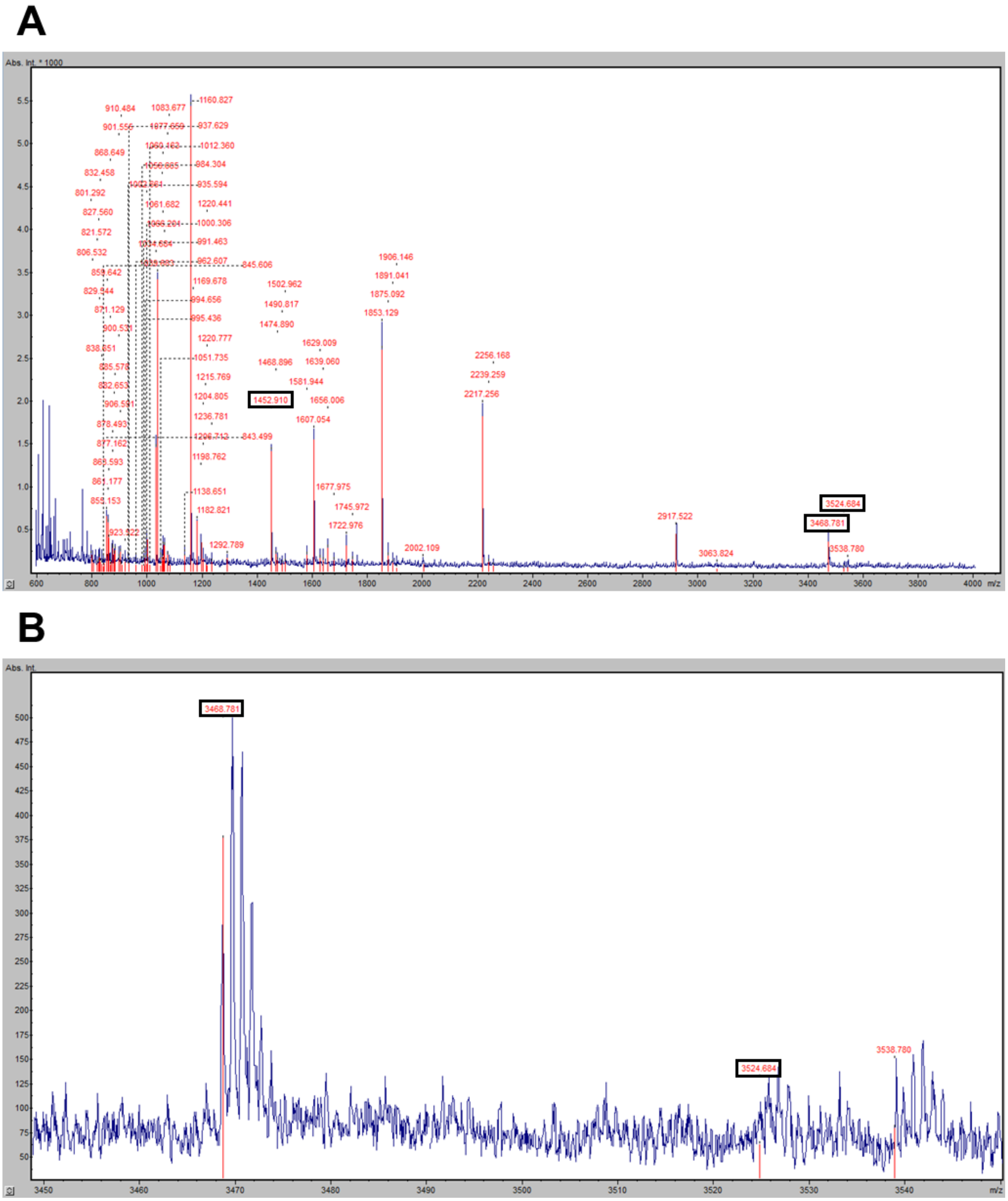


**Supplemental Fig. 1. Mass spectrometry data obtained on MCRα peptides**. **(A)** Full spectrum **(B)** Close up of the 3450-3550 m/z region. Peaks of interest are highlighted with boxes: $N^1$-methylhistidine275 containing peptide (1452.9 Da vs predicted mass of 1452.7 Da), Gln418 containing peptide (3468.8 Da vs predicted mass of 3468.6 Da). The alkylated peptide containing the thioglycine463 (3524.7 Da vs predicted mass of 3525.6 Da) is detected at a signal over noise threshold of 1.9. Low signal strength might be explained by increased instability of larger peptides.

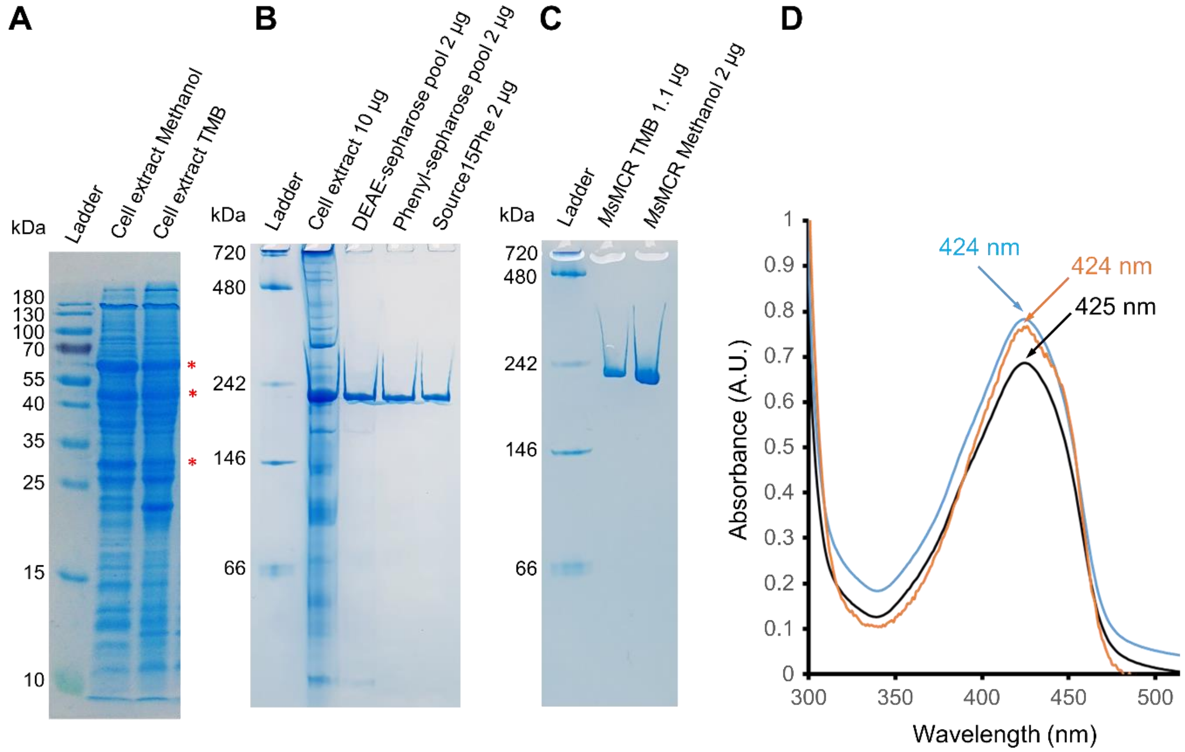


**Supplemental Fig. 2. Native PAGE and UV/visible spectra profile of purified *Ms*MCRs**. (**A**) SDS-PAGE of cell extract obtained from methanol and 3,4,5-trimethoxybenzoate (TMB) grown cells. The red stars show the position of the expected three MCR subunits. (**B**) High-resolution clear native PAGE of *Ms*MCR from TMB-grown cells for each purification step. (**C**) High-resolution clear native PAGE of purified *Ms*MCR from TMB and methanol-grown cells. (**D**) UV/visible spectra of purified *Ms*MCR from TMB-grown cells at a concentration of 5.6 mg/ml (in 50 mM Tris/HCl pH 7.6, 10% v/v glycerol and 2 mM dithiothreitol) under anaerobic atmosphere (black line) and after one hour $O_2$-exposition (blue line). The orange line corresponds to the UV/visible spectra of aerobically purified *Ms*MCR from methanol-grown cells at a concentration of 5.6 mg/ml (in 25 mM Tris/HCl pH 7.6, 10% v/v glycerol and 2 mM dithiothreitol). The peak at 424-425 nm is typical for a Ni(II) red1-silent inactive state.

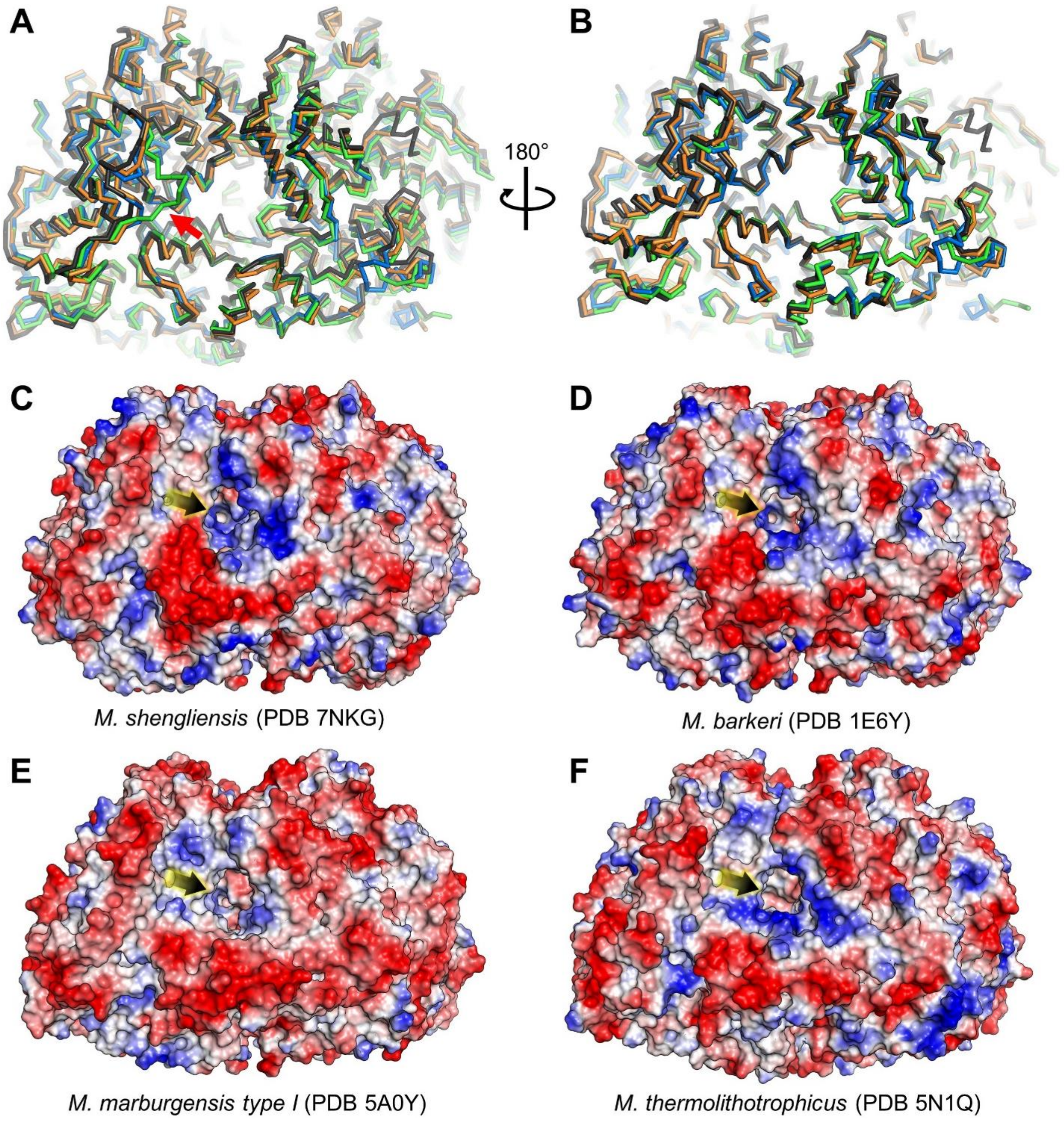


**Supplemental Fig 3. Structural and electrostatic charge differences between *Ms*MCR and its homologues**. (**A-B**) Superposition of the α-subunit of *Ms*MCR with MCR homologues. The Cα-trace is represented in ribbon with a color code corresponding to the different homologues: *Ms*MCR (PDB 7NKG) in green, MCR from *M. barkeri* (*Mb*MCR, PDB 1E6Y) in blue, MCR type I from *M. marburgensis* (*Mm*MCR, PDB 5A0Y) in orange and MCR from *M. thermolithotrophicus* (*Mt*MCR, PDB 5N1Q) in black. The deviating loop is highlighted with a red arrow. The difference between panel (**A**) and (**B**) is a 180° rotation along the y-axis. (**C-F**), Electrostatic charge surface representation. The charges from negative to positive are reflected by a color gradient from red to blue, respectively. An arrow points towards the active site channel entrance.

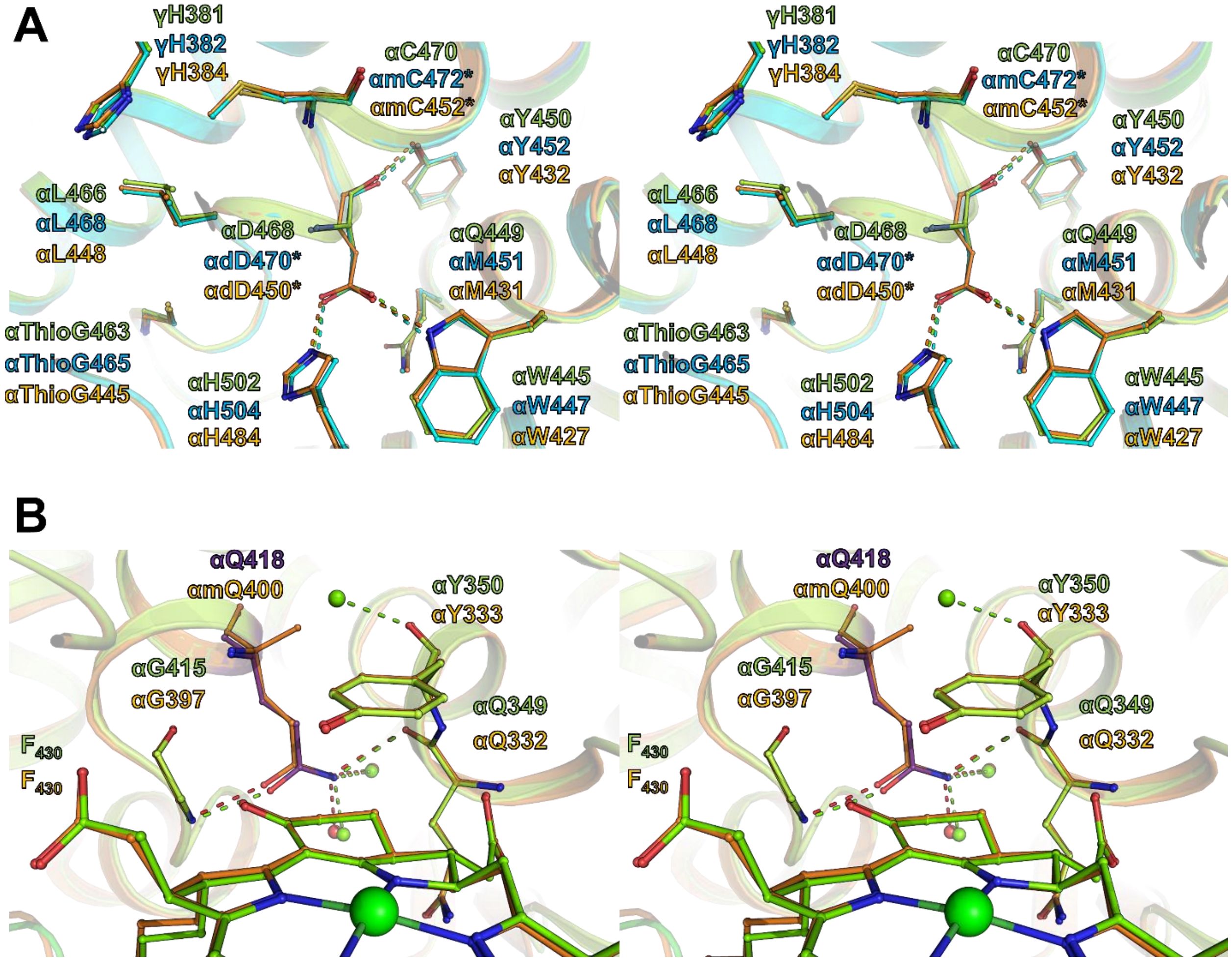


**Supplemental Fig 4. Close up of the environment at the expected modified residues didehydroaspartate, S-methylcysteine and 2(S)-methylglutamine in *Ms*MCR.** (**A**) Stereo-view of the close surrounding at the didehydroaspartate (αdDXXX*) and S-methylcysteine (αmCXXX*) positions. Both residues and their close surrounding are shown as balls and sticks and secondary structures are shown in transparent cartoon. *Ms*MCR, *Mb*MCR (PDB 1E6Y) and *Mm*MCR (PDB 5A0Y) are colored in green, cyan and orange, respectively. The only observable mutation in the close surrounding of the expected didehydroaspartate (*i.e.* αD468 in *Ms*MCR) is the methionine that turns to a glutamine (*i.e.* αM451 in *Mb*MCR, αM431 in *Mm*MCR, αQ449 in *Ms*MCR). (**B**) Stereo-view of the close surrounding at the 2(S)-methylglutamine (αmQ400 in *Mm*MCR) position. 2(S)-methylglutamine and its close surrounding is shown as balls and sticks and secondary structures are shown in transparent cartoon. *Ms*MCR, and *Mm*MCR are colored in green and orange, respectively. αQ418 in *Ms*MCR is highlighted in deep purple. The nickel from the $F_{430}$ cofactor is shown as a large green ball.

| Organism (PDB) | Sequence identity (%) | | | R.m.s.d (aligned Cα) | | |
|---|---|---|---|---|---|---|
| | α-subunit | β-subunit | γ-subunit | α-subunit | β-subunit | γ-subunit |
| *M. shengliensis* (7NKG) | 100 | 100 | 100 | 0 | 0 | 0 |
| *M. barkeri* (1E6Y) | 72.97 | 70.9 | 81.38 | 0.371 (477) | 0.411 (385) | 0.276 (205) |
| *M. marburgensis* type I (5A0Y) | 67.73 | 61.63 | 58.37 | 0.410 (424) | 0.655 (382) | 0.546 (192) |
| *M.thermolithotrophicus* (5N1Q) | 62.98 | 57.01 | 51.59 | 0.522 (448) | 0.716 (399) | 0.805 (217) |

**Supplemental Table 1. Sequence identity between the subunits of different MCRs and root mean square deviation (r.m.s.d.) of structurally characterized MCRs.**

**References regarding the substrate utilisation of methanogens and MCR post-translational modifications used in the phylogenetic tree (Fig. 1C).**

*Methanosarcina barkeri* [1,2]

*Methanosarcina mazei* [3]

*Methanimicrococcus blatticola* [4]

*Methanolobus profundi* [5]

*Methanomethylovorans hollandica* [6]

*Candidatus* Methanoperedens nitroreducens [7]

*Methermicoccus shengliensis* [8,9]

*Methanothrix thermoacetophila* [10]

*Methanocella conradii* [11]

*Methanosphaerula palustris* [12]

*Methanoculleus horonobensis* [13]

*Methanoplanus limicola* [14]

*Methanothermobacter marburgensis* [15,16]

*Methanothermobacter wolfeii* [16-18]

*Methanobrevibacter smithii* [19]

*Methanopyrus kandleri* [2,20]

*Methanotorris formicicus* [21,22]

*Methanocaldococcus vulcanius* [23]

*Methanothermococcus thermolithotrophicus* [22,24]

*Methanococcus maripaludis* [25]

ANME-1 from Black sea mats, Uncultured archaeon ANME-1 [26]

## References


1. Bryant, M.P.; Boone, D.R. Emended description of strain MST (DSM800T), the type strain of *Methanosarcina barkeri*. *Int J Syst Bacteriol* **1987**, *37*, 169-170, doi:10.1099/00207713-37-2-169.
2. Grabarse, W.; Mahlert, F.; Shima, S.; Thauer, R.K.; Ermler, U. Comparison of three methyl-coenzyme M reductases from phylogenetically distant organisms: unusual amino acid modification, conservation and adaptation. *J Mol Biol* **2000**, *303*, 329-344, doi:10.1006/jmbi.2000.4136.
3. Mah, R.A. Isolation and characterization of *Methanococcus mazei*. *Curr Microbiol* **1980**, *3*, 321-326, doi:10.1007/Bf02601895.
4. Sprenger, W.W.; van Belzen, M.C.; Rosenberg, J.; Hackstein, J.H.P.; Keltjens, J.T. *Methanomicrococcus blatticola* gen. nov., sp. nov., a methanol- and methylamine-reducing methanogen from the hindgut of the cockroach *Periplaneta americana*. *Int J Syst Evol Micr* **2000**, *50*, 1989-1999, doi:10.1099/00207713-50-6-1989.
5. Mochimaru, H.; Tamaki, H.; Hanada, S.; Imachi, H.; Nakamura, K.; Sakata, S.; Kamagata, Y. *Methanolobus profundi* sp. nov., a methylotrophic methanogen isolated from deep subsurface sediments in a natural gas field. *Int J Syst Evol Micr* **2009**, *59*, 714-718, doi:10.1099/ijs.0.001677-0.
6. Lomans, B.P.; Maas, R.; Luderer, R.; den Camp, H.J.M.O.; Pol, A.; van der Drift, C.; Vogels, G.D. Isolation and characterization of *Methanomethylovorans hollandica* gen. nov., sp. nov., isolated from freshwater sediment, a methylotrophic methanogen able to grow on dimethyl sulfide and methanethiol. *Appl Environ Microb* **1999**, *65*, 3641-3650.
7. Raghoebarsing, A.A.; Pol, A.; van de Pas-Schoonen, K.T.; Smolders, A.J.P.; Ettwig, K.F.; Rijpstra, W.I.C.; Schouten, S.; Damste, J.S.S.; Op den Camp, H.J.M.; Jetten, M.S.M., et al. A microbial consortium couples anaerobic methane oxidation to denitrification. *Nature* **2006**, *440*, 918-921, doi:10.1038/nature04617.
8. Cheng, L.; Qiu, T.L.; Yin, X.B.; Wu, X.L.; Hu, G.Q.; Deng, Y.; Zhang, H. *Methermicoccus shengliensis* gen. nov., sp. nov., a thermophilic, methylotrophic methanogen isolated from oil-production water, and proposal of *Methermicoccaceae* fam. nov. *Int J Syst Evol Microbiol* **2007**, *57*, 2964-2969, doi:10.1099/ijs.0.65049-0.
9. Mayumi, D.; Mochimaru, H.; Tamaki, H.; Yamamoto, K.; Yoshioka, H.; Suzuki, Y.; Kamagata, Y.; Sakata, S. Methane production from coal by a single methanogen. *Science* **2016**, *354*, 222-225, doi:10.1126/science.aaf8821.
10. Kamagata, Y.; Mikami, E. Isolation and characterization of a novel thermophilic *Methanosaeta* strain. *Int J Syst Bacteriol* **1991**, *41*, 191-196, doi:10.1099/00207713-41-2-191.
11. Lu, Z.; Lu, Y.H. *Methanocella conradii* sp. nov., a thermophilic, obligate hydrogenotrophic methanogen, isolated from chinese rice field soil. *Plos One* **2012**, *7*, doi:ARTN e3527910.1371/journal.pone.0035279.
12. Cadillo-Quiroz, H.; Yavitt, J.B.; Zinder, S.H. *Methanosphaerula palustris* gen. nov., sp. nov., a hydrogenotrophic methanogen isolated from a minerotrophic fen peatland. *Int J Syst Evol Micr* **2009**, *59*, 928-935, doi:10.1099/ijs.0.006890-0.
13. Shimizu, S.; Ueno, A.; Tamamura, S.; Naganuma, T.; Kaneko, K. *Methanoculleus horonobensis* sp. nov., a methanogenic archaeon isolated from a deep diatomaceous shale formation. *Int J Syst Evol Micr* **2013**, *63*, 4320-4323, doi:10.1099/ijs.0.053520-0.

14. Wildgruber, G.; Thomm, M.; König, H.; Ober, K.; Ricchiuto, T.; Stetter, K.O. *Methanoplanus limicola*, a plate shaped methanogen representing a novel family, the methanoplanaceae. *Arch Microbiol* **1982**, *132*, 31-36, doi:10.1007/Bf00690813.
15. Fuchs, G.; Stupperich, E.; Thauer, R.K. Acetate assimilation and the synthesis of alanine, aspartate and glutamate in *Methanobacterium thermoautotrophicum*. *Arch Microbiol* **1978**, *117*, 61-66, doi:10.1007/BF00689352.
16. Wagner, T.; Kahnt, J.; Ermler, U.; Shima, S. Didehydroaspartate Modification in Methyl-Coenzyme M Reductase Catalyzing Methane Formation. *Angew Chem Int Ed Engl* **2016**, *55*, 10630-10633, doi:10.1002/anie.201603882.
17. Winter, J.; Lerp, C.; Zabel, H.P.; Wildenauer, F.X.; König, H.; Schindler, F. *Methanobacterium wolfei*, sp. nov., a new tungsten-requiring, thermophilic, autotrophic methanogen. *Syst Appl Microbiol* **1984**, *5*, 457-466, doi:10.1016/S0723-2020(84)80003-X.
18. Wasserfallen, A.; Nölling, J.; Pfister, P.; Reeve, J.; de Macario, E.C. Phylogenetic analysis of 18 thermophilic *Methanobacterium* isolates supports the proposals to create a new genus, *Methanothermobacter* gen. nov., and to reclassify several isolates in three species, *Methanothermobacter thermautotrophicus* comb. nov., *Methanothermobacter wolfeii* comb. nov., and *Methanothermobacter marburgensis* sp. nov. *Int J Syst Evol Micr* **2000**, *50*, 43-53, doi:10.1099/00207713-50-1-43.
19. Miller, T.L.; Wolin, M.J.; Demacario, E.C.; Macario, A.J.L. Isolation of *Methanobrevibacter smithii* from human feces. *Appl Environ Microb* **1982**, *43*, 227-232, doi:10.1128/Aem.43.1.227-232.1982.
20. Kurr, M.; Huber, R.; König, H.; Jannasch, H.W.; Fricke, H.; Trincone, A.; Kristjansson, J.K.; Stetter, K.O. *Methanopyrus kandleri*, gen. and sp. nov. represents a novel group of hyperthermophilic methanogens, growing at 110 °C. *Arch Microbiol* **1991**, *156*, 239-247, doi:10.1007/Bf00262992.
21. Takai, K.; Nealson, K.H.; Horikoshi, K. *Methanotorris formicicus* sp. nov., a novel extremely thermophilic, methane-producing archaeon isolated from a black smoker chimney in the Central Indian Ridge. *Int J Syst Evol Micr* **2004**, *54*, 1095-1100, doi:10.1099/ijs.0.02887-0.
22. Wagner, T.; Wegner, C.E.; Kahnt, J.; Ermler, U.; Shima, S. Phylogenetic and structural comparisons of the three types of methyl coenzyme M reductase from *Methanococcales* and *Methanobacteriales*. *J Bacteriol* **2017**, *199*, doi:10.1128/JB.00197-17.
23. Jeanthon, C.; L'Haridon, S.; Reysenbach, A.L.; Corre, E.; Vernet, M.; Messner, P.; Sleytr, U.B.; Prieur, D. *Methanococcus vulcanius* sp. nov., a novel hyperthermophilic methanogen isolated from East Pacific Rise, and identification of *Methanococcus* sp. DSM 4213(T) as *Methanococcus fervens* sp. nov. *Int J Syst Bacteriol* **1999**, *49*, 583-589, doi:10.1099/00207713-49-2-583.
24. Huber, H.; Thomm, M.; König, H.; Thies, G.; Stetter, K.O. *Methanococcus thermolithotrophicus*, a novel thermophilic lithotrophic methanogen. *Arch Microbiol* **1982**, *132*, 47-50, doi:10.1007/Bf00690816.
25. Jones, W.J.; Paynter, M.J.B.; Gupta, R. Characterization of *Methanococcus maripaludis* sp. nov., a new methanogen isolated from salt marsh sediment. *Arch Microbiol* **1983**, *135*, 91-97, doi:10.1007/Bf00408015.
26. Shima, S.; Krüger, M.; Weinert, T.; Demmer, U.; Kahnt, J.; Thauer, R.K.; Ermler, U. Structure of a methyl-coenzyme M reductase from Black Sea mats that oxidize methane anaerobically. *Nature* **2012**, *481*, 98-101, doi:10.1038/nature10663.
